# PReS-FINAL-2082: Reliability and responsiveness of the standardized universal pain evaluations for rheumatology providers for children and youth (SUPER-KIDZ)

**DOI:** 10.1186/1546-0096-11-S2-P94

**Published:** 2013-12-05

**Authors:** N Luca, J Stinson, BM Feldman, S Benseler, D Beaton, AM Bayoumi

**Affiliations:** 1 Paediatric Rheumatology, Alberta Children's Hospital, Calgary, Canada; 2 Hospital for Sick Children, Toronto, Canada; 3 Paediatric Rheumatology, Hospital for Sick Children, Toronto, Canada; 4 Mobility Program Clinical Research Unit, Toronto, Canada; 5 Internal Medicine, Li Ka Shing Knowledge Institute of St Michael's Hospital, Toronto, Canada

## Introduction

Pain is the most common symptom in children and youth with juvenile idiopathic arthritis (JIA), however, currently there is no comprehensive validated pain measure for this population. The Standardized Universal Pain Evaluations for Rheumatology Providers for Children and Youth (SUPER-KIDZ) is a new multi-dimensional online pain tool, developed to fill this gap in clinical care.

## Objectives

To determine the test-retest reliability and responsiveness of the computerized 20-item version of the SUPER-KIDZ pain tool in children with JIA.

## Methods

A single center prospective cohort study of JIA patients aged 8-18 years was performed. The SUPER-KIDZ questionnaire was administered to children expected to have stable pain for test-retest reliability analysis of each item using intra-class correlation coefficients (ICC) and weighted Cohen's kappa. Responsiveness of each SUPER-KIDZ item to change in pain was evaluated in patients undergoing intra-articular steroid injection(s) who are expected to have improvement in pain. Measures of responsiveness included standardized response mean (SRM), Wilcoxon signed rank test, linear mixed model regression, and receiver operating characteristic (ROC) curve analysis. Internal consistency of the three SUPER-KIDZ subscales (sensory, interference, emotional) was measured using ordinal reliability alpha and item-total correlation.

## Results

Fifty-one children were included, of which 40 (78%) were female, and had a median of 3 active joints (1-5) and median physician global assessment of 2.5 cm (1.5-4) on 10 cm visual analog scale. Internal consistency was acceptable (ordinal α = 0.73-0.92) for the sensory, interference, and emotional SUPER-KIDZ subscales. Good test-retest reliability (ICC or weighted kappa ≥0.80) was found for 15 SUPER-KIDZ items in at least one analysis. Reliability was strongest for the items on pain intensity, pain frequency, pain duration and physical function, and weakest for questions related to sleep, having fun, catastrophizing, and feeling angry. At 2 weeks post-injection, 16 items were responsive to change in pain (SRM = 0.66-0.82, significant Wilcoxon signed rank and/or linear mixed model regression). ROC curve analysis of 9 items gave an area under the curve of ≥0.70, adequately distinguishing between improved and unimproved subjects. The questions less responsive to change in pain were those related to fatigue frequency and emotional function (feeling angry, cheerful, worried).

## Conclusion

The majority of items of the new online SUPER-KIDZ tool have excellent test-retest reliability and responsiveness properties. The questions about fatigue and emotional function are less responsive to change after a joint injection procedure and could be tested after a cognitive intervention. If validity is demonstrated, this measure could be implemented as a standardized comprehensive pain tool for JIA patients, thereby fulfilling a longstanding gap in the care of patients with JIA.

## Disclosure of interest

None declared.

